# Simulation of the dynamics of primary immunodeficiencies in CD4+ T-cells

**DOI:** 10.1371/journal.pone.0176500

**Published:** 2017-04-27

**Authors:** Gabriel N. Teku, Mauno Vihinen

**Affiliations:** Department of Experimental Medical Science, Lund University, Lund, Sweden; South Texas Veterans Health Care System, UNITED STATES

## Abstract

Primary immunodeficiencies (PIDs) form a large and heterogeneous group of mainly rare disorders that affect the immune system. T-cell deficiencies account for about one-tenth of PIDs, most of them being monogenic. Apart from genetic and clinical information, lots of other data are available for PID proteins and genes, including functions and interactions. Thus, it is possible to perform systems biology studies on the effects of PIDs on T-cell physiology and response. To achieve this, we reconstructed a T-cell network model based on literature mining and TPPIN, a previously published core T-cell network, and performed semi-quantitative dynamic network simulations on both normal and T-cell PID failure modes. The results for several loss-of-function PID simulations correspond to results of previously reported molecular studies. The simulations for TCR PTPRC, LCK, ZAP70 and ITK indicate profound changes to numerous proteins in the network. Significant effects were observed also in the BCL10, CARD11, MALT1, NEMO, IKKB and MAP3K14 simulations. No major effects were observed for PIDs that are caused by constitutively active proteins. The T-cell model facilitates the understanding of the underlying dynamics of PID disease processes. The approach confirms previous knowledge about T-cell signaling network and indicates several new important proteins that may be of interest when developing novel diagnosis and therapies to treat immunological defects.

## Introduction

The human immunome consists of the genes and proteins essential both for the innate and adaptive immunity. Interactions between these proteins are indispensable for immune responses [1]. Studies have been carried out to identify and characterize the essential immunome interactome, i.e. the totality of interactions in the immune system [1, 2]. Knowledge from these studies enables the investigation of the dynamic behavior of networks in both health and disease. The immunome interactome varies depending on the cell-type, timing and localization of expressed and active proteins.

CD4+ T-cells are crucial immune response white blood cells. They recognize and bind to antigens on antigen-presenting cells via the cell surface T-cell receptor (TCR) complex [3]. Antigen binding to the TCR triggers a sequence of signaling events that lead to the activation and nuclear transportation of specific transcription factors (TFs) [3]. In the nucleus, these TFs transactivate genes that are required for T-cell responses. CD4+ T-cells are divided into subpopulations of T helper 1 (Th1), Th2, Th17, regulatory T (Treg) and follicular helper T (Tfh) cells [4]. Each cell type plays different roles in the immune response by virtue of their different master regulator TFs and signature cytokine expression [5].

Here, we investigated the qualitative dynamics of the naïve CD4+ T-cells in both health and in disease in primary immunodeficiencies. Protein interaction networks in T-cells and their role in various diseases have been investigated [6, 7]. Primary immunodeficiencies (PIDs) are intrinsic diseases of the immune system, and are typically rare with heterogeneous phenotypes. Currently about 300 PIDs are known. Disease-causing variants in PIDs have been collected into the IDbases [8] and other databases and are available for more than 150 PIDs. Differential diagnosis of PIDs can be difficult due to overlapping signs and symptoms. Several classification schemes have been made, including the frequently updated classification by the International Union of Immunological Societies (IUIS) expert committee for PIDs [9]. PIDs have also been classified with a network approach that clusters the diseases based on signs, symptoms and laboratory parameters [10]. The severity of PIDs ranges from mild to moderate, and severe to lethal. By integrating the diverse information sources, systems level studies of the underlying mechanisms on PIDs can be conducted.

In systems biology, the reconstruction of cellular networks and their simulations facilitate studies of diseases as perturbations (or alterations) to the networks [11, 12]. These approaches provide insight on the dynamics of biomolecular interactions that drive cellular processes and contribute towards deciphering biological processes in both health and disease. Disease-causing variations can affect protein-protein interaction (PPI) networks at the cellular or tissue level. Studies of quantitative dynamics of PPIs require kinetic parameters and reaction constants. A problem emerges as reaction constants for most of the reactions have not been determined. Further, these network calculations are very computer intensive. The number of parameters, even for a moderate size network is so large that calculations would be very costly and time-consuming. Another approach amenable to larger networks of few tens to hundreds of nodes is to use qualitative and semi-quantitative dynamic methods [13–15], which provide useful models for approximating systems.

In this study, we employed a semi-quantitative method, the normalized HillCube Boolean approach [16], to simulate the dynamics during the activation of naïve CD4+ T-cells. With these simulations, we investigated the mechanisms of perturbations of known PID-causing proteins and revealed novel putative PID-related factors. Semi-quantitative simulations with synchronous updates were performed, and *in silico* validated. The simulations qualitatively replicated PIDs due to variations in PID-related proteins which disrupt essential signal transduction pathways during T-cell development from pre- to mature CD4+ T-cells [12]. Further, several novel proteins affected by PIDs were identified.

## Results

### The naïve CD4+ T-cell activation network

**Fig 1:**
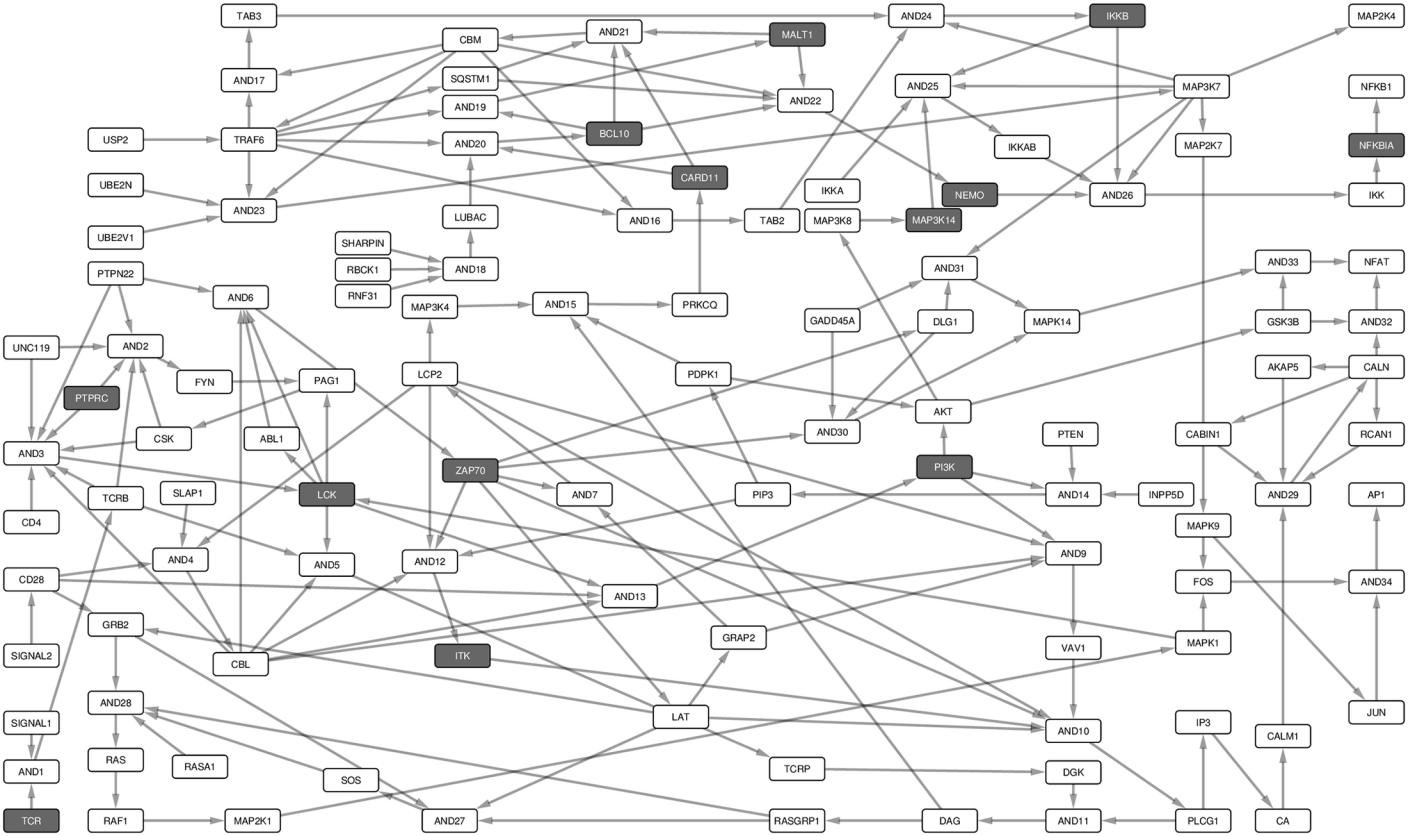
Naïve CD4+ T-cell activation Boolean network model. The network consists of 182 links and 118 nodes (including Boolean operators), 19 of which are input nodes, i.e., no link points to them (S1 Table). The Boolean network represents the naïve CD4+ T-cell activation events. The boxes represent non-PID (white) and PID proteins (gray). Spheres denote the AND gate. Activating links have a pointed head and solid line while inhibiting links have a blunt head and dashed line. Signal 1 represents peptide-MHC/TCR complex while Signal 2 represents co-receptor-ligand association, e.g. CD80-B7. Since the network focuses on TCR/CD28 signaling events, some events, e.g. for survival signaling that occur after antigen mediated T-cell activation and response through interleukin 2 (IL2), have not been fully considered.

We reconstructed the signal transduction network for naïve CD4+ T-cells by using the T cell PPI network, TPPIN [17] as a basis for formulating reaction equations. The nodes and links of the PPI network were used to mine the published literature for valid reaction equations on CD4+ T-cells. The TPPIN is a PPI network that contains 227 core signal transduction interactions derived from integrated, time series, gene expression data sets. This network does not include link directions and, in most cases, lacks cellular context. Thus, we mined manually the direction, interaction and cellular context information by literature survey. We included only signaling interactions that were TCR/CD28-dependent and CD4+ T-cell-specific, leaving 85 interactions, which were used for reconstructing the Boolean network model (S1 Table). The interactions were defined manually as Boolean equations using the sum-of-product (SOP) form. The SOP representation offers a convenient means to represent Boolean equations of a signaling network in a hypergraph [18]. Proteins, i.e. the nodes, represent the Boolean variables. The edges (hyperarcs) represent the interactions between proteins and are signed either activating (+) or inhibiting (-). Edges have a tail that begins from a start node and a head (or arrow), which points to an end node, indicating the direction of signal transduction. Multiple edges with the same end node were summed by an OR operator. The AND gate was used as a product operator for multiple incoming edges that together are required to activate or inhibit a protein. 19 input nodes did not have in-coming links (Fig 1).

We started by analyzing the structure of the network and the signaling paths between the initial events of the TCR-dependent activation and the late events that involve the activation of the major TFs that turn on the expression of response genes. The TCR complex, its co-receptor CD4, and the co-stimulatory receptor CD28, are involved in the initial events, while the TFs AP1, NFAT, and NF-κB control the late events of T-cell activation [3].

The TCR is activated when it binds to an antigen (signal 1) presented by an antigen presenting cell. Another signal (signal 2) through the co-activation receptor CD28 is needed to elicit activation, survival and response [19]. The multiple paths from receptors to TFs guarantee a fail-safe and robust T-cell activation [20, 21]. It may also imply that the sensitivity of the level of activation is modulated by different routes [22]. On the other hand, signal transduction may be critical if only a single route exists from the receptors, through the network, to the TFs.

**Fig 2:**
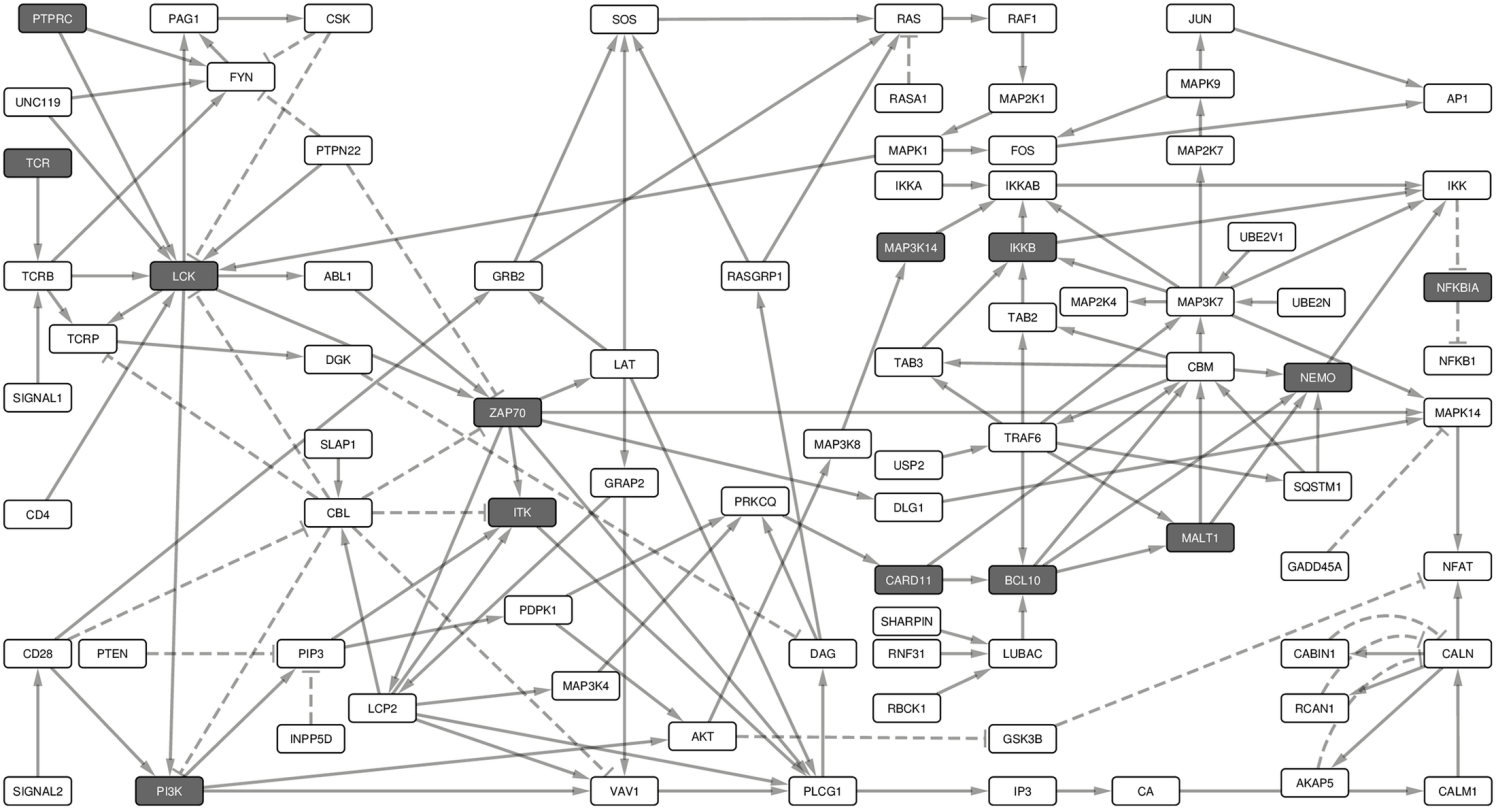
Boolean model transformed into its underlying interaction graph. The network consists of nodes and links derived from the Boolean network model without the AND operator. The interaction graph consists of 85 nodes and 146 links, and represents the underlying interaction network of the model. The nodes are as described in Fig 1. The network shows the paths through which signals from the receptors are channeled through the network to the TFs, which turn on the response genes.

**Fig 3:**
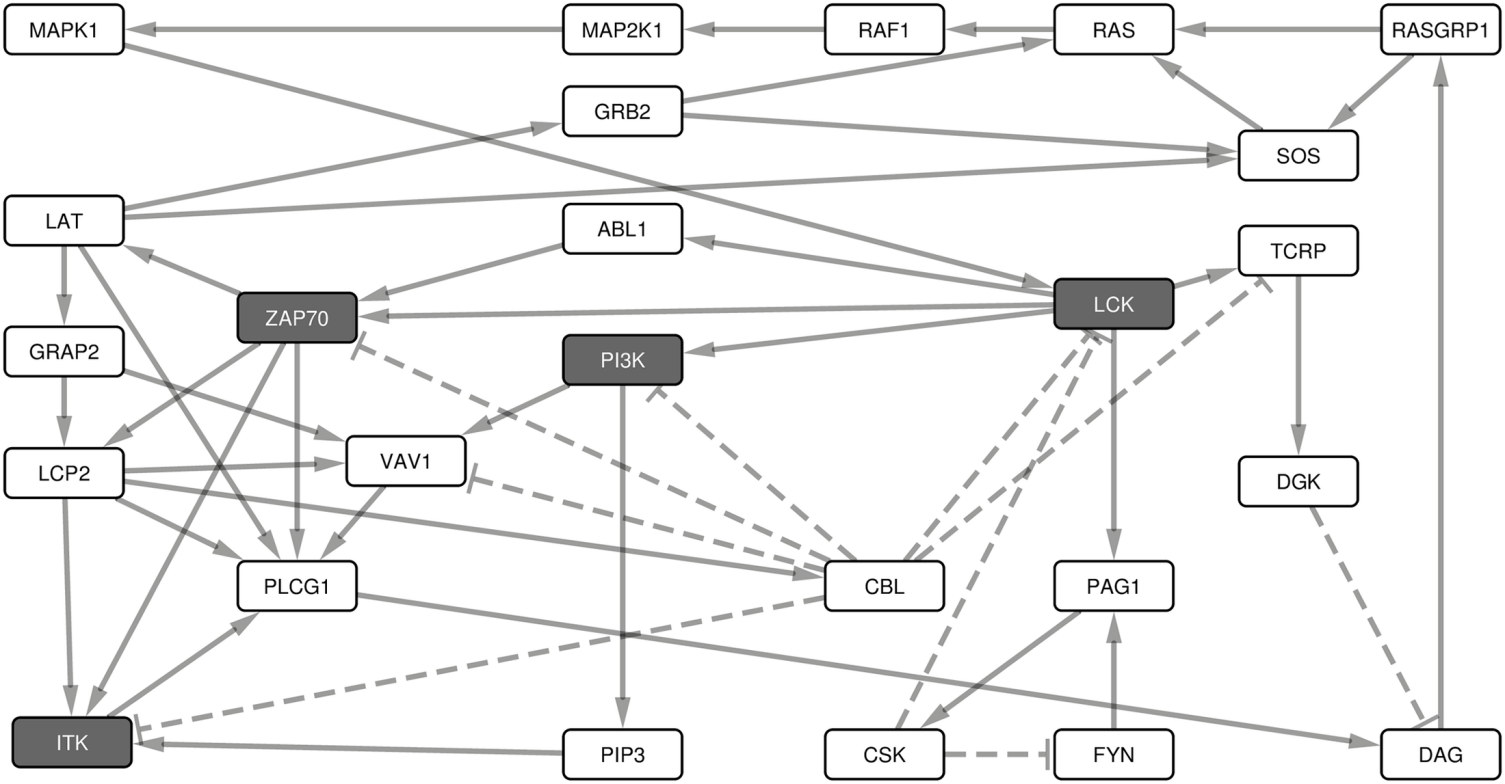
The strongly connected components of the interaction graph. The strongly connected component of the interaction graph consists of 25 nodes and 48 links. This subnet shows the interconnectedness and cross-talk of the early signals after the antigen-TCR ligation.

We identified signaling paths from signals 1 and 2 to the major response TFs NF-κB, AP1 and NFAT. For this purpose, we converted the Boolean network into an interaction network (Fig 2). Such a network captures the dependencies, interactions, and thus, the paths through which signals are transduced through the network. The interaction network consists of a connected component with 85 nodes interconnected by 146 links. To detect the part of the network with the most cross-talk between the signaling pathways, we identified the strongly connected component that consists of 25 nodes and 48 links (Fig 3).

**Fig 4:**
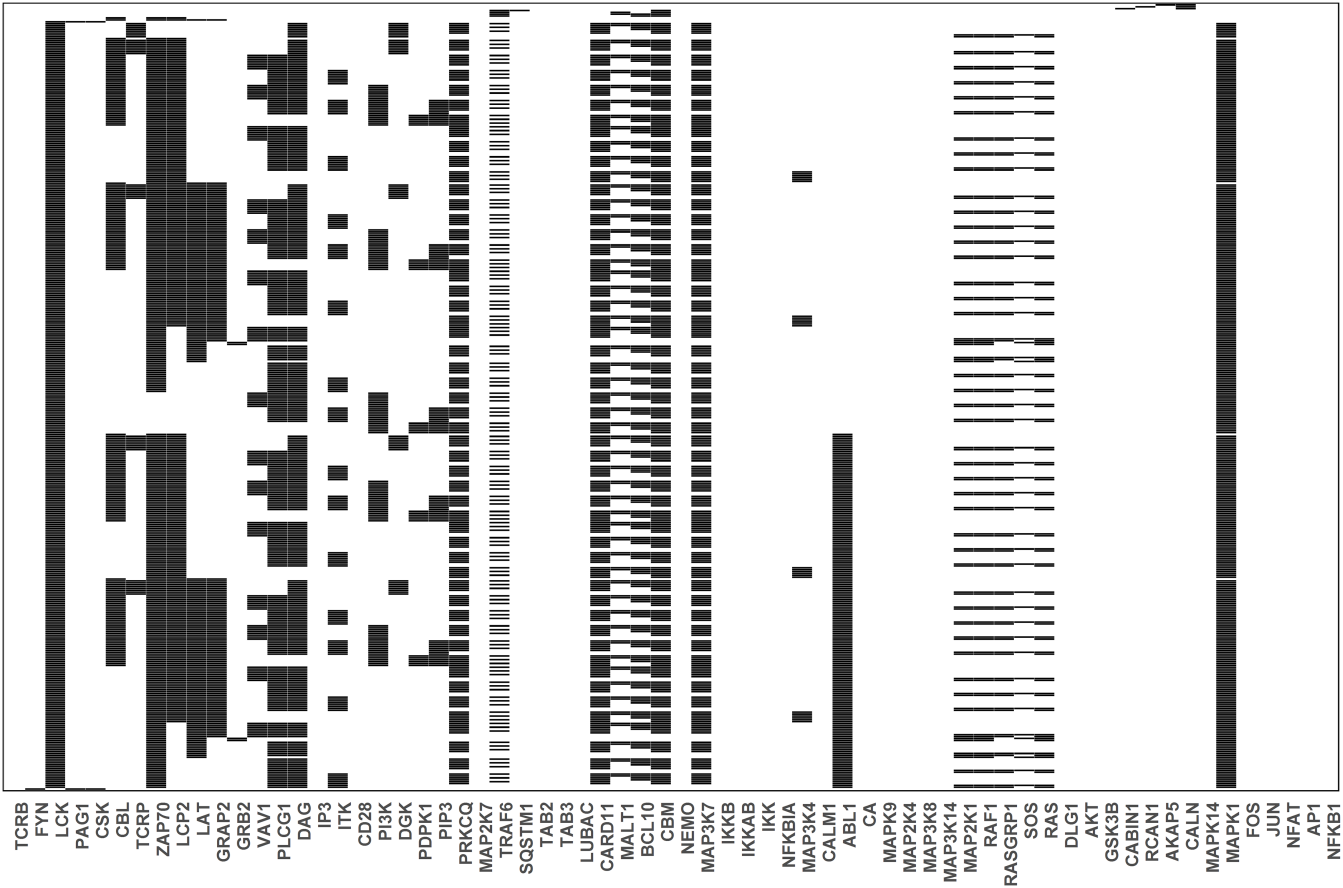
Feedback loops or cycles in the interaction graph. Signaling paths having FBLs from signals 1 and 2 to the major transcription factors identified from the interaction graph. The columns represent the Boolean update equations and are labeled with the updated protein. Each row represents an FBL, and consists of the proteins located along it. On each row, cells with a black background indicate proteins that are along the FBL. There are 419 loops, containing on average 14 proteins.

To identify proteins essential for signal transduction from the receptors to the downstream actuators we analyzed the feedback loops (FBLs) in the network. Those proteins whose Boolean update equations are along most of the FBLs are considered essential. Input and output nodes were not included in the FBLs. We identified 419 such loops, of which the longest spans 20 nodes and the shortest 2 nodes (Fig 4). The median and mean length of the FBLs is 14 nodes long. Among the PID proteins, LCK was in 409 FBLs, ZAP70 in 380, CBM in 316, CARD11 in 312, BCL10 in 210, ITK in 120, PI3K in 110 and MALT1 in 106 FBLs. The other PID proteins, NEMO, IKKB, NFKBIA and MAP3K14, do not occur in any of the FBLs. PTPRC is an input node and is thus not included in any of the FBLs.

### Validation of reconstructed network and identification of the wild type attractor

After the engagement of the TCR complex and the co-activator CD28, a series of signal transduction cascades occur in naïve CD4+ T-cells [23] and are captured by the reconstructed network. The signaling cascades lead to response either via NF-κB, AP1 or NFAT [3]. The reconstructed network is cogent if the major TFs (here TFs NF-κB, AP1 and NFAT that together activate IL2) and the signaling components that lead to their activation are turned on.

To ensure that the reconstructed model reproduces CD4+ T-cell activation, we performed simulations by iteratively changing the initial states of the input nodes while making sure that the network represented the main signaling events. We used normalized HillCube dynamic simulations [16] with signals 1 and 2 turned on and validated the simulations *in silico*. This network model was used for the subsequent analyses. Additionally, we performed simulations by turning signal 1 on and signal 2 off, and vice versa. When either of the signals were turned off, only AP1 and NFAT, but not NF-κB, were activated.

**Fig 5:**
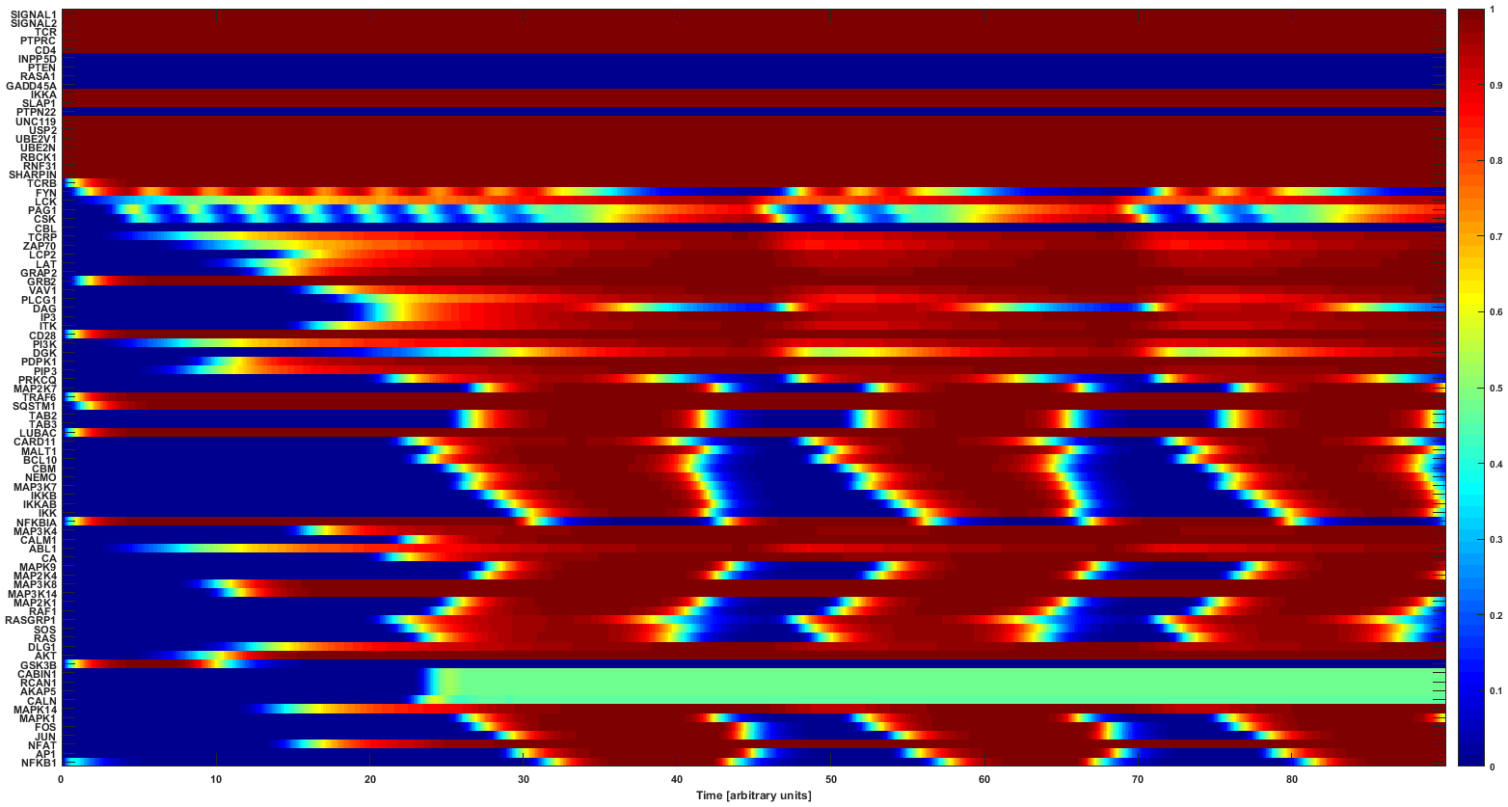
Attractor basin of the CD4+ T-cell network model normalized HillCube simulation. The basin of attractors of the CD4+ T-cell network model simulated using the normalized HillCube algorithm. The horizontal axis denotes time in arbitrary units.

The normalized HillCube simulations were run until the networks reached their attractor states. The model settled in a cyclic attractor or limit cycle after about 40 update rounds (arbitrary time units, Fig 5). The network subsequently continues in a cycle attractor after about every 20 updates. This attractor is in accordance with published experimental results [3, 24], which also is evident in the activation of the major downstream TFs (AP1, NF-κB, and NFAT) when signals 1 and 2 are turned on.

### PID failure analysis

To study the effects of disease-causing variations on the long-term dynamics of naïve CD4+ T-cells, we perturbed PID proteins in the network model and simulated their dynamics with the normalized HillCube update approach. Twelve PIDs are known to affect the proteins in the network including BCL10, CARD11, IKKB, ITK, LCK, MALT1, MAP3K14, NEMO, NFKBIA, PI3K, PTPRC, TCR (TRAC, a component of the TCR complex) and ZAP70. The proteins were identified from the ImmunoDeficiency Resource [25], IDbases [8], the most recent classification by the IUIS expert committee for PIDs [9] and a recent review [26]. These proteins are expressed at the pre-CD4+ stage during T-cell development and differentiation. The effects of knockouts or overexpression of these proteins to the signaling pathways were investigated by turning them off (on) during simulation. The resulting perturbed attractors were probed for differences compared to the wild type attractor.

**Fig 6:**
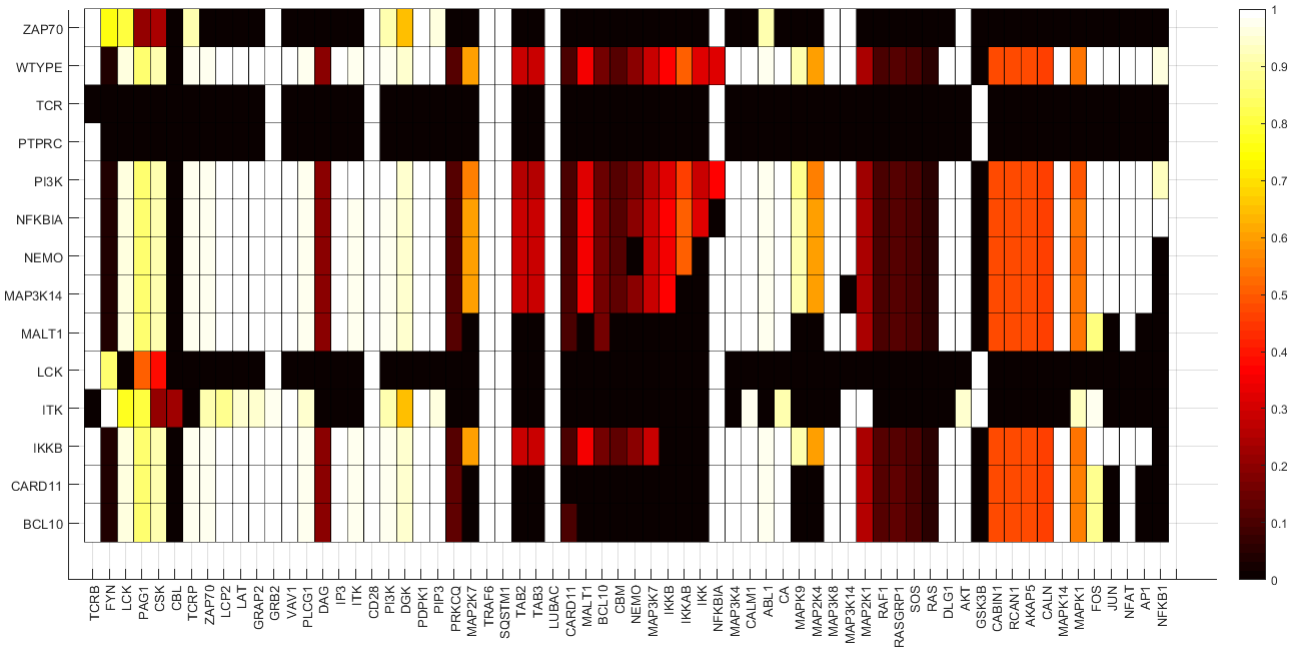
Wild type and PID attractors of the CD4+ T-cell network simulation. The node states for the wild type and the PID-perturbed attractors (knockout perturbation of LCK, ZAP70, ITK, IKKB, NEMO, CARD11, MALT1, BCL10, NFKBIA, PTPRC, MAP3K14 and knockin perturbation of PI3K) attractors. The attractors are represented by the rows while the states of the nodes in the attractors are represented on the columns. The state of a node for an attractor is represented by the color of the cell on the row of the attractor; black means inactive whereas white means activate.

The three major TF pathways were dysregulated in the attractors of PIDs involved in the early events of the TCR-dependent T-cell activation including ITK, LCK, PTPRC, TCR and ZAP70 perturbations (Fig 6). AP1 was inactive in the PID attractors for BCL10, CARD11, ITK, LCK, MALT1, MAP3K14 and PTPRC. The NF-κB pathway was dysregulated in all the PID-perturbed attractors, except that for PI3K and NFBIA. The NFKBIA knockout and the PI3K overexpression simulations were identical to the wild type. The perturbations indicate profound effects in the networks for almost all the PIDs. Several novel proteins were found to be affected by the complete and partial knockouts (knockins) of the PID proteins.

### Correlation to PID severity

The severity of PIDs varies greatly from very mild to life-threatening conditions. Severe combined immunodeficiency (SCID) is associated with high susceptibility to bacterial, viral and fungal infections [27]. Persistent infections with respiratory and gastrointestinal viruses and opportunistic pathogens are frequent and often associated with protracted diarrhea and failure to thrive. According to the IUIS classification [9], most of the PIDs in this study are associated with SCIDs with reduced numbers or absent T and B cells. These include BCL10, CARD11, IKKB, ITK, LCK, MALT1, MAP3K14, NEMO, PTPRC, TCR and ZAP70 deficiencies. Interestingly, the attractors for these proteins show severe dysregulation (Fig 6).

Gain-of-function variants in the *PIK3CD* gene, a catalytic subunit of the PI3K heterodimeric complex is associated with a milder PID [28–30]. Additionally, variants in the gene that code for NFKBIA are associated with various forms of ectodermal dysplasia with immunodeficiency (EDA-ID) [31–34]. The attractors for these two proteins are very similar to the wild type form.

### Novel PID-associated proteins

The discovery and cataloging of the PIDs is an ongoing effort. With the improvement, development and reduction in the cost of new technologies, more PIDs are identified. Due to the large number, rarity and overlapping symptoms of PIDs, the diagnosis may be late, difficult and costly. Several efforts have been made to ease diagnosis by classifying PIDs [9, 10], predicting and prioritizing candidate genes and proteins [35–38]. The FBLs of our model and the PID-perturbed attractors from simulations provide information about proteins that affect several pathways and could be involved with PIDs. Proteins which are along at least 20 FBLs include the majority of the investigated PIDs and several proteins essential for CD4+ T cell activation and functions. Interestingly, most of these proteins also indicate abrogated signaling in the attractors for most of the PIDs. To evaluate, *in silico*, the effects of perturbing the non-PIDs in Table 1, we performed knockout simulations for each node, except for CBL for which knockin simulation was performed, as CBL is turned off in the wild type attractor. Twenty-one (70%) of the perturbed nodes are impaired in TCR-dependent T cell activation. Further, we investigated the Human Gene Connectome (HGC) (ref) and found that many of the proteins involved in numerous FBLs have significant connections to known PID proteins. Taken together, the genes coding for these proteins are worth considering when prioritizing genes during challenging diagnosis.

**Table 1:** Number of FBLs along which each protein is in the T cell network model.

Protein	No of FBLs	Effect on NFAT pathway	Effect on NF-κB pathway	Effect on AP1 pathway	BRP	Core proteins^b^
LCK	409	0	0	0		
MAPK1	404	1	1	1	0.00275	NFKBIA (PI3K, PTPRC)
ZAP70	380	0	0	0		
DAG	344	1	0	0		
CBM^a^	316	1	0	0		
PRKCQ	312	1	0	0	0.00024	IKKB (CARD11, LCK, MALT1, NEMO, PTPRC, ITK, PI3K, NFKBIA)
CARD11	312	1	0	0		
MAP3K7	312	1	0	0	0.00281	IKKB (CARD11, MALT1, NEMO, NFKBIA, ZAP70, ITK, PI3K)
LCP2	310	1	0	0	0.00048	ITK (LCK, ZAP70, PTPRC, PI3K, NFKBIA, IKKB)
PLCG1	304	1	0	0	0.00167	ITK (PI3K, LCK, ZAP70)
BCL10	210	1	0	0		
LAT	193	1	0	0	0.00119	ZAP70 (PTPRC, ITK, PI3K, LCK, NFKBIA)
CBL	190	0	0	0	0.00114	ITK (PI3K, ZAP70, LCK, PTPRC)
ABL1	189	0	0	0	0.00633	NFKBIA (LCK, ZAP70, ITK, PI3K)
GRAP2	171	1	0	0	0.00048	ITK (PTPRC, PI3K, NFKBIA, IKKB, MALT1)
TRAF6	160	1	0	0	0.00191	NFKBIA (MALT1, IKKB, CARD11, LCK, NEMO, ZAP70, PI3K)
VAV1	120	1	0	0	0.00036	ITK(PI3K, LCK, ZAP70, NFKBIA)
ITK	120	0	0	0		
PI3K	110	1	1	1		
MALT1	106	1	0	0		
MAP2K1	92	1	1	1	0.00072	PI3K (ITK, NFKBIA)
RAF1	92	1	1	1	0.00329	PI3K (LCK, ITK, NFKBIA)
RAS	92	1	1	1	0.00335	LCK (ZAP70, PI3K, NFKBIA, IKKB)
RASGRP1	86	1	1	1	0.00125	ZAP70 (PI3K, IKKB, MALT1, CARD11, LCK, ITK, PTPRC, NEMO)
PIP3	70	1	0	0		
SOS	47	1	1	1	0.00036	ITK (PI3K, LCK, ZAP70, CARD11)
TCRP	40	1	1	1		
DGK	40	1	1	1	0.00556	NFKBIA (ZAP70, CARD11)
PDPK1	30	1	0	0	0.00102	MALT1 (CARD11, PI3K, IKKB, NFKBIA, LCK, NEMO, ZAP70)
MAP3K4	24	1	0	0	0.01673	PI3K (LCK, IKKB, NEMO)

Rows with tan background are for PIDs.

^a^CBM deficiency is considered as a PID because it is a complex, all of whose components are related to PIDs.

^b^The first core protein is the most significant to the target and those in parenthesis are other significant ones for the target (BRP < 0.05).

## Discussion

In this study, we used the normalized HillCube approach to simulate the PID knockout effects in the naïve CD4+ T-cell network dynamics. To achieve this, a network was reconstructed based on evidence from the literature and a previously identified core T-cell network. By using normalized HillCube simulations, we refined and *in silico* validated the reconstructed network. The normalized HillCube perturbation studies qualitatively replicated complete loss-of-function variation effects for several PIDs at CD4+ T-cell developmental stages.

Comparison of the wild type to the PID attractors highlighted significant differences in the signal transduction patterns for ITK, LCK, PTPRC, TCR and ZAP70. The effects of the LCK, PTPRC, TCR and ZAP70 perturbations are severe. Knockout simulations for these proteins qualitatively capture major changes in signaling patterns. The differences between the wild type and MAP3K14, NEMO and IKKB PID simulations were somewhat minor. In the BCL10, MALT1, CARD11, MAP3K14, NEMO and IKKB knockouts, the NF-κB pathway was the most affected. This is because these proteins connect receptor-dependent signals to the distal NF-κB pathway [24]. Knockout of any of these genes may cause the IKK complex, the major NF-κB regulator, to be impaired, leaving NFKBIA bound to NFKB1, and preventing its nuclear transportation and function as a TF [24]. These results show that our approach of simulating effects of protein variations in networks is effective when the affected proteins are in the core of the interconnected network or along non-redundant paths belonging to crucial pathways. No major changes were revealed in overexpression perturbation, as in PI3K, or redundant signaling path between the receptor to the TF. In the NFKBIA PID heterozygous variants that either lack the phosphorylation sites [31, 32] or truncate the protein [34] protect it from phosphorylation-induced proteosomal degradation. The inactivated NFKBIA sequesters NFKB1 in the cytosol [32]. Thus, the deficient NFKBIA acts as a dominant negative form for NFKB1, reducing NFKB1’s activity and causing the reduction of TCR activation-dependent cytokine response [32]. Indeed, there were no observed differences between the PI3K and NFKBIA PID simulations compared to the wild type. LCK and ZAP70 perturbations that cause major effects are present in over 90% of the FBLs in the network and CARD11 and the CBM complex in 75% of the FBLs. Seven of the 12 PID proteins emerge in the FBLs, most of which are proximal TCR activation events, highlighting the fact that the simulation studies are effective for detecting effects of centrally located proteins.

Antigen-TCR complex ligation causes conformational alterations of CD3 chains, which contain immunoreceptor tyrosine-based activation motifs (ITAMs) on which they are phosphorylated by LCK [3]. This is an essential step in early TCR activation. The LCK kinase activity is regulated by the antagonistic actions of the membrane protein tyrosine phosphatase PTPRC and the carboxy-terminal Src kinase (CSK) (30). The phosphorylation of Tyr505 in LCK by CSK inhibits LCK activity via auto-phosphorylation of Tyr394 in the catalytic domain. The dephosphorylation of the Tyr505 by PTPRC relieves this inhibition [39]. TCR is crucial for T cell activation and cytokine response, and simulation of TCR deficiency shows profound impairment of all TF pathways. A homozygous variant of TRAC, a crucial component of the TCR complex, causes this deficiency [40]. The deficiency is associated with lymphadenopathy, recurrent infections and hepatosplenomegaly. Because the increased activity of LCK is crucial for the T cell response after antigen stimulation, the PTPRC knockout causes a severe perturbation. This is confirmed by disease-causing variations in the gene [41–45]. The known variants include large deletions [44] and amino acid substitutions [45]. Immunodeficiencies caused by the lack of LCK activity lead to T cells that are low in number and non-responsive, which in turn causes susceptibility to infections. Our PTPRC-perturbed simulations indicate that all the signaling paths of NFAT, NFKB1 and AP1 TFs, crucial for TCR-dependent response, are disrupted.

The activation of LCK is a crucial early step for T-cell activation and response. The phosphorylation of the CD3 ITAMs leads to the recruitment of ZAP70 and its activation by LCK. ZAP70 subsequently phosphorylates LAT, leading to the formation of the LAT signalosome (the proximal signaling complex) [46]. LAT signalosome transduces signals to pathways that are indispensable for the three major TFs necessary for T-cell activation and response. Thus, the improper constitution of this signaling site affects multiple pathways and disrupts the transduction of TCR activation signals, as verified by our simulations.

The absence of LCK signaling disrupts the NFAT pathways and abrogates the T-cell response. The LCK deficiency is associated to naive CD4+ T-cell lymphopenia, respiratory tract infections, and early-onset autoimmune inflammation [47–49]. The major effects of this PID on naïve CD4+ T-cells are a profoundly defective TCR signaling, lack of calcium/magnesium signaling and defective NF-κB response. Our simulation of the knockout perturbation confirms the dysregulation of most signaling events associated with the calcium signaling, thereby affecting the AP1, NFKB1 and NFAT signaling pathways. LCK and ZAP70, the two vital components necessary for the formation of the LAT signalosome, are turned off in the LCK-perturbed attractor. This suggests that the LAT signalosome is disrupted and thus, downstream signaling is impaired. As shown in Fig 6, the signaling components required for the AP1, NF-κB, NFAT family of proteins, including the calcium-dependent signaling, are turned off in the LCK knockout attractor. The affected signaling components include PLCG2, PIP2, IP3, DAG and CALN.

Because of the proximity of ZAP70 to LCK in the early activated TCR signaling events, the effects of ZAP70 are expected to be similar. This is indeed the case. Partially affected signaling occurs in ZAP70 deficiency, but downstream responses, like proliferation, are abrogated because of the TCR signaling defect. Severe conditions caused by the ZAP70 deficiency have been diagnosed in several patients [50–55]. Like the PTPRC and LCK knockout simulations, the major effectors associated to the calcium signaling are turned off in the ZAP70 perturbed attractor. Based on these results, the activated T-cells would become anergic and/or undergo apoptosis. SYK, the ZAP70 homolog in non-T-cells, is expressed at high levels in the CD4+ T-cells of ZAP70-deficient patients [50, 53]. The SYK expression might compensate for the lack of ZAP70, and has been used to explain the less severe phenotype of the ZAP70 deficiency [50].

During the constitution of the LAT signalosome, LCP2 and PLCG1 bind to LAT and are phosphorylated by ZAP70 [46]. The phosphorylated LCP2 then recruits ITK, which leads to the activation of PLCG1. PLCG1 hydrolyzes its substrate PIP2 to generate second messengers, IP3 and DAG. ITK is a non-receptor tyrosine kinase expressed in T-cells and has been described as an important component of proximal TCR signaling [56].

Several homozygous ITK variants cause PID [57–60]. The ITK deficiency is associated with naive CD4+ T-cell lymphopenia, modest change in the number of CD4+ T-cells, impaired positive and negative selection of thymocytes due to reduced TCR signal levels, recurrent infections (for example, herpes virus infections), autoimmune cytopenias, lymphoproliferation, lymphadenopathy and hepatosplenomegaly. Genotype studies point to a twofold increase in activated CD4+ T-cells, impaired activation-induced cell death and decreased levels of TCR signaling. Additionally, there is evidence that TXK could substitute for ITK [61]. The lack of ITK in mice is mitigated by the ability of TXK to activate PLCG1 [62]. ITK is present in both the strongly connected component and several (29%) of the FBLs. These findings indicate that in the absence of ITK, T-cells are activated, but signaling resulting from TCR stimulation leads to impaired response. Indeed, the attractors from our perturbed simulations showed abrogation of the NFAT, AP1 and NF-κB pathways. This agrees with normal, but progressive decrease in T cell numbers that may be caused by defective response in the TCR-dependent response pathways, which are indispensable for IL-2 transactivation and T cell response [63, 64].

The constitution of the CBM complex is an essential event in the regulation of NF-κB pathway. After the TCR/CD28 activation, PRKCQ is activated and recruited to the proximal signalosome. Here, PRKCQ activates CARD11 [65], which leads to its association with BCL10. Because BCL10 is constitutively bound to MALT1, the association of CARD11 to BCL10 leads to the formation of the CBM complex. Several PIDs have been connected to variations that occur on the genes that code for CARD11 [66], MALT1 [67] and BCL10 [68]. The CARD11 PID case is caused by a homozygous premature stop codon on the gene that codes for CARD11, and truncates its kinase-like domain. A homozygous variant in the CARD domain of *MALT1* causes MALT1 PID. The known BCL10 PID case is due to a homozygous splice-site variation at intron 1 of the gene encoding BCL10. The CARD11 PID is associated with hypogammaglobulinemia, severe interstitial pneumonia, dyspnea and respiratory tract infections [66]. The MALT1 deficiency is associated with bronchiectasis, mastoiditis, chronic aphthous ulcers, gastritis, gingivitis, duodenitis and meningitis while the BCL10 PID is associated with hypogammaglobulinemia, gastroenteritis, otitis, respiratory tract infection and several viral infections [68]. Although the CBM PIDs show normal T cell counts, the BCL10 and MALT1 deficiencies show predominantly naïve CD4+ T cells, including severely abrogated TCR-dependent NF-kB signaling and cytokine response [69]. As expected, the pathways for NF-κB and AP1 are severely disrupted in the attractors of the CARD11, MALT1 and BCL10 PIDs.

The major regulator of NF-κB is the IKK complex [24]. It consists of two protein kinases, IKKA and IKKB and a regulatory protein, NEMO [70]. The activation of the IKK complex is NEMO-dependent. After the TCR/CD28 activation PRKCQ is activated and recruited to the proximal signalosome, where it activates CARD11 [65], which leads to the formation of the CBM complex. The TRAF6 oligomerizes with the CBM complex through the association with MALT1 and BCL10 [71]. This oligomerization recruits UBE2V1 which polyubiquitinates and, thus, activates TRAF6 [72]. The activated TRAF6 in turn activates MAP3K7, which subsequently coordinates the assembly of the IKK complex [71, 73].

Some PIDs have been linked to both IKKB and NEMO [74–77]. A complete loss of function homozygous truncating variant, a duplicating variant, and a nonsynonymous nucleotide substitution on the gene that codes for IKKB have been reported to cause the disease [77–79]. IKKB deficiency is associated with life-threatening bacterial, fungal, and viral infections, defective immunoglobulin production and hypo- or agammaglobulinemia. Although T cell numbers are normal, T cell subsets are lower, and peripheral T cells fail to respond to stimulation. *IKBKB* loss of function variants abrogates signaling and response via the NF-κB pathway in these patients [24]. Genetic studies have revealed several PID cases linked to *IKBKG*, the gene that codes for NEMO [74, 75, 80–82]. The disease results from amino acid substitution and exon skipping variations. The NEMO deficiency is associated with anhidrotic ectodermal dysplasia, polysaccharide non-response, various infectious diseases, colitis, ectodermal dysplasia, conical teeth, variable defects of skin pigmentation and monocyte dysfunction [74, 75]. The T cell counts are normal but TCR activation is impaired, especially NF-κB activation. In accordance with these studies, our simulation indicates that the NEMO and IKKB perturbations lead to inactivation of NF-κB, despite normal activation of AP1 and NFAT [24, 83, 84].

MAP3K14 is a member of the family of mitogen-activated protein kinases that is involved in both the canonical [24] and non-canonical [85] NF-κB pathways. In the canonical NF-κB pathway, the CD28 co-stimulatory signal is required for the MAP3K14 activation through MAP3K8 (COT). After activation by AKT1, MAP3K8 activates MAP3K14, which in turn contributes in the activation and subsequent ubiquitination of NFKBIA [71, 73, 86]. The ubiquitination of NFKBIA releases NFKB1 which is translocated into the nucleus and results in T-cell response. In the non-canonical NF-κB pathway, MAP3K14 associates with IKKA to induce the phosphorylation and subsequent ubiquitination of the p100 subunit [85, 87]. This leads to the proteolysis of NFKB2/p100 to NFKB2/p52-RELB dimer, which is translocated to the nucleus and transactivates κB-containing genes for response [85].

A PID caused by a biallelic variation in the gene coding for MAP3K14 protein leads to loss of its kinase activity [88]. This variant disrupts both the canonical and non-canonical NF-κB pathways in immune response cell-types [88]. Despite the normal overall T cell numbers, several T cell subsets show defective response and perturbation. The MAP3K14 PID is associated with several microbial infections, including bacterial and viral infections [88]. The MAP3K14 PID-perturbed simulations are in accordance with its crucial and non-redundant role in T cells as seen in the defective activation of NFKB1, albeit normal activation of AP1, NFAT and MAPK14 [89].

The results for simulations of NFKBIA and PI3K did not differ from wild type. To investigate the effects of variants and knockouts in these proteins, dedicated networks would be needed with more information about downstream factors.

Our results show PID-caused trends in the cellular dynamics of the CD4+ T-cells when the affected proteins are involved in non-redundant paths along major TF signaling pathways. The downstream signaling events show minor effect on the network dynamics than the early events. This paper is the first attempt, as far as we are aware, to investigate, with systems biological simulations, the effects of variations in immune response proteins in PIDs. We found profound effects in the ITK, LCK, PTPRC, TCR and ZAP70 perturbed simulations, and less profound but noticeable effects in the BCL10, CARD11, IKKB, MALT1, MAP3K14 and NEMO perturbed simulations.

The non-PID proteins in Table 2 are indispensable for T cell activation and response, are affected in several of the simulated PID attractors and have also been associated with other diseases. Several of them have been identified as candidate PIDs. *VAV1*, *RAF1*, *LAT*, *LCP2* and *MAPK1* were identified as candidate PID genes with high confidence by Keerthikumar et al. [37]. Moreover, 15 out of 22 of the proteins are predicted to be candidates by another recent study [38]. These include LCP2, CBL, TRAF6, MAP3K7, VAV1, PLCG1, PRKCQ, RAF1, ABL1, PDPK1, GRAP2, LAT, MAPK1, MAP3K4 and MAP2K1. Several of the candidate genes are central in the Human Gene Connectome (Table 2) providing independent proof for their significance. As the connectome is not complete, the fact that there is no support from this method does not mean that our findings were not significant even from this point of view.

**Table 2:** Tuned parameters of nodes in the Odefy-simulated T cell network model.

Influenced node	Influencing node(s)	τ	n	k
PAG1	[]^a^	1	20	0.9
PAG1	[]	1	20	0.9
DAG	DGK	1	20	0.9
DGK	[]	1	20	0.9
DGK	[]	1	3	0.9
DGK	[]	1	3	0.9
LCK	MAPK1	10	20	0.1
CBL	[]	3	20	0.9
CALN	CABIN1	1	3	0.9
CALN	RCAN1	1	3	0.9
CALN	AKAP5	1	3	0.9

^a^All influencing nodes.

PAG1, phosphoprotein membrane anchor with glycosphingolipid microdomains 1; DAG, second messenger, diacylglycerol; DGK, diacylglycerol kinases; LCK, LCK proto-oncogene, Src family tyrosine kinase; MAPK1, mitogen-activated protein kinase 1 (ERK); CBL, Cbl proto-oncogene; CALN, calcineurin complex; CABIN1, calcineurin Binding Protein 1, RCAN1, regulator of calcineurin 1, AKAP5, A-kinase anchoring protein 5.

Nine of the proteins in Table 2 are protein kinases (MAPK1, PRKCQ, MAP3K7, PLCG1, LAT, MAP2K1, RAF1, PDPK1, MAP3K4), 4 are mitogen-activated protein kinases (MAPK1, MAP2K1, RAF1 and MAP3K4), 3 are serine-threonine kinases (PRKCQ, MAP3K7, and PDPK1), and 3 have guanyl-nucleotide exchange factor activity (VAV1, RASGRP1 and SOS). Four of the proteins are linked to various forms of the Noonan syndrome (CBL, MAP2K1, RAF1 and SOS), 5 to various types of tumors (MAPK1, PRKCQ, ABL1, GRAP2 and RAS) and one to an autoimmune disorder (RASGRP1). Seven of the genes are not linked to any disease (MAP3K7, LCP2, LAT, VAV1, DGK, PDPK1 and MAP3K4). The listed proteins are strong PID candidates; however, their involvement in PIDs needs to be experimentally verified. In the case of the NFKBIA perturbed simulations we observed local effect on NF-κB and for PI3K, no effects. Further simulation studies of these PIDs will require more specific networks, if applicable.

Several studies suggest candidate PID genes [35, 37, 38]. Ortutay and Vihinen constructed a PPI network of immune system-specific proteins, proteins with high network statistics and PID-related Gene Ontology term enrichment scores [35]. Itan and Casanova identified the top 1% of genes that were biologically close to known PIDs and, and from these selected the ones with similar Gene Ontology terms as the known PIDs [38]. A machine learning technique, support vector machine, was applied by Keerthikumar and colleagues to identify candidate PIDs by utilizing binary features from PIDs and non-PIDs [37]. The above approaches were successful in identifying several candidate genes that were subsequently verified to be PID related. Our approach focuses on T-cell-specific PIDs and how they affect other components of the cellular signaling dynamics. This, as well as other evidence presented above, allowed us to identify the candidate PIDs.

Diagnosis and prognosis of PIDs is still often problematic. Our approach provides novel insights into the mechanisms of PID effects on signaling cascades and may highlight novel targets for therapy downstream of the defective proteins. The presented approach can be used to study PIDs of any cellular system and even diseases outside the immune system.

## Methods

### Network reconstruction and analysis

The T-cell PPI network (TPPIN), a core network of PPIs specific to T-cells [17], was used as the basis for extensive literature survey and the reconstruction of the Boolean equations for the T cell model. Only those nodes that have been demonstrated to play a crucial role in the TCR/CD28-dependent activation of CD4+ T cells were retained.

The CellNetAnalyzer version 2016.1 [18] was used for identifying feedback loops in the underlying interaction graph of the model. The base R software version 3.2.3 [90] and Cytoscape version 3.3.0 [91] were used for data analysis and network visualization, respectively. The strongly connected components were calculated using *igraph*, a library for network and graph analyses in R [92].

A Boolean model consists of N nodes/proteins *X*_*1*_, *X*_*1*_,…, *X*_*N*_. The proteins are represented by variables *x*_i_ that take values {0, 1} [93]. Each protein, *x*_i_ is influenced by a set of proteins *R*_*i*_ = *{X*_*1*_, *X*_*1*_,…, *X*_*N*_*}* connected to it. Based on the values of their influencing proteins *R*_*i*_, for each time step, the value of each protein *x*_i_, is calculated from the update function *B*:*{0*, *1*^*N*^*}*. Because the time is discretized in Boolean simulations, at time point *t + 1*, updates are done synchronously as follows [93, 94],

$$x_{i}\left(t+1\right)=B_{i}\left(x_{i1}\left(t\right),\;x_{i2}\left(t\right),\ldots ,\;x_{iN_{i}}\left(t\right)\right)\;\epsilon \;\{0,\;1\},\;i=1,\;2,\;\ldots ,\;N.$$

The Boolean update functions, *B*_*i*_, are converted into a system of continuous ordinary differential equation (ODE) model where *x*_*i*_ takes values [0, 1] using the following ODE equation

$${\dot{x}}_{i}\;=\frac{1}{\tau_{i}}\left({\bar{B}}_{i}\;({\bar{x}}_{i1},\;{\bar{x}}_{i2},\ldots ,\;{\bar{x}}_{iN_{i}}\right)-\;{\bar{x}}_{i}),$$

where, ${\bar{B}}_{i}$ is a continuous homologue of the discrete function *B*_*i*_, parameter τ_*i*_ represents the life-time of the protein, and ${\bar{x}}_{i}$ describes its decay.

Odefy [16], a toolbox compatible with MATLAB, transforms *B*_*i*_ to the ODE system and computes the solution of the system using the BooleCubes [95] as follows,

$${\bar{B}}^{I}\left({\bar{x}}_{1},\;{\bar{x}}_{2},\ldots ,\;{\bar{x}}_{N}\right)=\;\sum_{x_{1}=0}^{1}\sum_{x_{1}=0}^{1}\ldots \sum_{x_{N}=0}^{1}\left[B\left(x_{1},\;x_{2},\;\ldots ,\;x_{N}\right).\prod_{i=1}^{N}\left(x_{i}{\bar{x}}_{i}\;+\left(1-x_{i}\right)\left(1-{\bar{x}}_{i}\right)\right)\right].$$

${\bar{B}}^{I}$, the BooleCube, is obtained from the multilinear interpolation of the Boolean update function *B*_*i*_. Biomolecular interactions show switch-like behavior and are modeled using sigmoidal functions. Thus, the Hill function, $f\left(\bar{x}\right)=\;{\bar{x}}^{n}/\left({\bar{x}}^{n}+\;k^{n}\right)$, was used to smoothen the affine multilinear BooleCube, to obtain the sinusoidal HillCube [95]. Hence, the parameter *n* was introduced (the Hill coefficient or slope of the Hill function), to represent the cooperativity between the protein interactions and parameter *k* represent the value at which the activation is half-maximal. The HillCube is obtained from the BooleCube as follows,

$${\bar{B}}^{H}\left({\bar{x}}_{1},\;\ldots ,\;{\bar{x}}_{N}\right)=\;{\bar{B}}^{I}\left(f_{1}\left({\bar{x}}_{1}\right),\ldots ,f_{N}\left({\bar{x}}_{N}\right)\right).$$

To obtain perfect homologues of the Boolean update functions *B*_*i*_, the HillCube functions are normalized to the unit interval to give the normalized HillCube [95] as follows,

$${\bar{B}}^{Hn}\left({\bar{x}}_{1},\;\ldots ,\;{\bar{x}}_{N}\right)=\;{\bar{B}}^{I}\left(\frac{f_{1}\left({\bar{x}}_{1}\right)}{f_{1}\left(1\right)},\ldots ,\frac{f_{N}\left({\bar{x}}_{N}\right)}{f_{N}\left(1\right)}\right).$$

The network model used in this study is available in SBML qual format (S1 Text) on the website http://structure.bmc.lu.se/tcell_net/web_session/#/.

### Basin of attraction and attractor identification

The Odefy was used to simulate the qualitative dynamics of the network model. It provides simulation algorithms for both synchronous and asynchronous updates and allows simulations based on the BooleCubes [16]. We used the normalized HillCube functions, which represent the normalized BooleCubes in the range [0–1]. Boolean dynamic simulations were performed using normalized HillCube simulations [95]. Except for nodes involved in some negative feedback loops, the default parameter values were used. The default parameters for the normalized HillCube were *n* = 3, *k* = 0.5 and τ = 1. Table 2 lists non-default parameters for nodes on some feedback loops. The variable *n* represents the Hill exponent of the Hill function and is used for converting the discrete Boolean update functions that take value {0, 1} into their continuous BooleCube equivalents that have values [0, 1]. It captures the influence that nodes of the same Boolean equation have on each other. *k* is a variable to control the continuous relaxation of the Boolean step function. It represents the value at half-maximal activation of a protein. τ is a decay parameter; for each protein, the higher its value the slower the decay of the protein. The simulations were run until the network dynamics settled in an attractor.

### Perturbation

The Analysis of PID effects was performed for each protein encoded by a PID gene using the normalized HillCube simulations. For each perturbation, the node was converted to an input before assigning a state, either off or on, depending on the PID. For example, if the PID occurs as a result of over-activity of the protein, then the perturbed state is ON. This state was maintained until the simulation transitioned into the attractor. The parameter values used in the wild type simulations were maintained for all the PID perturbed simulations. The end result of the simulation represents the perturbed PID attractor.

### Primary immunodeficiency data

PID proteins expressed after the pre-CD4+ T-cell development stage were retrieved from the IDbases [8], the most recent updated IUIS expert committee classification of PID data [9], and a recent survey [26], and used for the PID failure mode simulations. The PIDs included LCK, ZAP70, ITK, IKKB, NEMO, CARD11, MALT1, BCL10, NFKBIA, PTPRC, MAP3K14 and PI3K deficiencies.

## Supporting information

S1 Table CD4+ T-cell activation Boolean network model update equations. The table lists Boolean equations of protein activation used in the network model and simulations.
(DOCX)

S1 Text SBML qual. The CD4+ T-cell network model in SBML qual format. The contains the CD4+ T-cell network qualitative model in the SBML qual format.
(SBML)
