# Supplementary material for: Simulation of the dynamics of primary immunodeficiencies in CD4+ T-cells
Source: PLoS One. 2017 Apr 27;12(4):e0176500. doi: 10.1371/journal.pone.0176500 (PMC5407609; doi:10.1371/journal.pone.0176500)
Supplement: S1 Table — The table lists Boolean equations of protein activation used in the network model and simulations. (DOCX) [file pone.0176500.s001.docx]

| **Species** | **Reactions** | **References** |
| --- | --- | --- |
| ABL1 | LCK | (1, 2) |
| AKAP5 | CALN | (3, 4) |
| AKT | PDPK1 \|\| PI3K | (5-7) |
| AP1 | FOS && JUN | (8) |
| BCL10 | TRAF6 && LUBAC && CARD11 | (9-12) |
| CA | IP3 | (13) |
| CABIN1 | CALN | (12) |
| CALM1 | CA | (14) |
| CALN | CALM1 & ~CABIN1 & ~RCAN1 & ~AKAP5 | (10, 15, 16) |
| CARD11 | PRKCQ | (9, 10, 17) |
| CBL | SLAP1 && LCP2 && ~CD28 | (18, 19) |
| CBM | MALT1 && CARD11 && BCL10 && SQSTM1 | (12, 20-22) |
| CD28 | SIGNAL2 | (23) |
| CD4 | INPUT | (23) |
| CSK | PAG1 | (24, 25) |
| DAG | PLCG1 && ~DGK | (26, 27) |
| DGK | TCRP | (26, 27) |
| DLG1 | ZAP70 | (28) |
| FOS | MAPK9 \|\| MAPK1 | (26) |
| FYN | TCRB && PTPRC && ~CSK && ~PTPN22 && UNC119 | (25, 29-31) |
| GADD45A | INPUT |  |
| GRAP2 | LAT | (26) |
| GRB2 | LAT \|\| CD28 | (24, 25, 32) |
| GSK3B | ~AKT | (33) |
| IKK | IKKAB && NEMO && MAP3K7 && IKKB | (12, 34, 35) |
| IKKA | INPUT |  |
| IKKAB | MAP3K7 && IKKA && IKKB && MAP3K14 | (12, 36) |
| IKKB | MAP3K7 && TAB2 && TAB3 | (12, 37) |
| INPP5D | INPUT |  |
| IP3 | PLCG1 | (23) |
| ITK | LCP2 && ZAP70 && PIP3 && ~CBL | (26, 38-40) |
| JUN | MAPK9 | (23, 41, 42) |
| LAT | ZAP70 | (43) |
| LCK | (PTPRC && CD4 && TCRB && UNC119 && ~CBL && ~PTPN22 && ~CSK) \|\| MAPK1 | (44-46) |
| LCP2 | GRAP2 && ZAP70 | (4, 47) |
| LUBAC | RBCK1 && RNF31 && SHARPIN | (12, 48, 49) |
| MALT1 | TRAF6 && TRAF6 | (9-11, 17) |
| MAP2K1 | RAF1 | (50) |
| MAP2K4 | MAP3K7 | (51) |
| MAP2K7 | MAK3K7 | (51) |
| MAP3K4 | LCP2 | (52) |
| MAP3K7 | TRAF6 && UBE2N && UBE2V1 && CBM | (17, 51, 53) |
| MAP3K8 | AKT | (54) |
| MAP3K14 | MAP3K8 | (35) |
| MAPK1 | MAP2K1 | (26, 55) |
| MAPK14 | ( ZAP70 && ~GADD45A && DLG1) \|\| ( MAP3K7 && ~GADD45A && DLG1) | (51, 56) |
| MAPK9 | MAP2K7 | (57, 58) |
| NEMO | CBM && BCL10 && MALT1 && SQSTM1 | (12, 59-61) |
| NFAT | (CALN && ~GSK3B) \|\| (MAPK14 && ~GSK3B) | (26, 33, 62, 63) |
| NFKB1 | ~NFKBIA | (26) |
| NFKBIA | ~IKK | (26) |
| OUTPUT | AP1 | (26) |
| OUTPUT | NFAT | (26) |
| OUTPUT | NFKB1 | (26) |
| PAG1 | FYN \|\| LCK | (45, 64) |
| PDPK1 | PIP3 | (65) |
| PI3K | LCK && CD28 && ~CBL | (18, 66) |
| PIP3 | PI3K && ~INPP5D && ~PTEN | (67, 68) |
| PLCG1 | VAV1 && ITK && LCP2 && ZAP70 && LAT | (24, 38, 69) |
| PRKCQ | PDPK1 && DAG && MAP3K4 | (10, 69-72) |
| PTEN | INPUT |  |
| PTPN22 | INPUT |  |
| PTPRC | INPUT | (23) |
| RAF1 | RAS | (73) |
| RAS | SOS && RASGRP1 && ~RASA1 && GRB2 | (74-78) |
| RASA1 | INPUT |  |
| RASGRP1 | DAG | (27, 79) |
| RBCK1 | INPUT |  |
| RCAN1 | CALN | (15) |
| RNF31 | INPUT |  |
| SHARPIN | INPUT |  |
| SIGNAL1 | INPUT | (23) |
| SIGNAL2 | INPUT | (23) |
| SLAP1 | INPUT |  |
| SOS | GRB2 && LAT && RASGRP1 | (80, 81) |
| SQSTM1 | TRAF6 | (82) |
| TAB1 | INPUT |  |
| TAB2 | TRAF6 && CBM |  |
| TAB3 | TRAF6 && CBM | (12) |
| TCR | INPUT | (12) |
| TCRB | SIGNAL1 && TCR | (23) |
| TCRP | TCRB && LCK && ~CBL | (23, 44, 46) |
| TRAF6 | USP2 \|\| CBM | (10, 83) |
| UBE2N | INPUT |  |
| UBE2V1 | INPUT | (17) |
| UNC119 | INPUT |  |
| USP2 | INPUT |  |
| VAV1 | LCP2 && GRAP2 && PI3K && ~CBL | (84) |
| ZAP70 | LCK && ABL1 && ~CBL && ~PTPN22 | (18, 23, 42, 46, 85) |

# References

1. Zipfel PA, Zhang W, Quiroz M, Pendergast AM. Requirement for Abl kinases in T cell receptor signaling. Current biology : CB. 2004;14(14):1222-31.

2. Wang JY. The capable ABL: what is its biological function? Mol Cell Biol. 2014;34(7):1188-97.

3. Coghlan VM, Perrino BA, Howard M, Langeberg LK, Hicks JB, Gallatin WM, et al. Association of protein kinase A and protein phosphatase 2B with a common anchoring protein. Science. 1995;267(5194):108-11.

4. Liu SK, Fang N, Koretzky GA, McGlade CJ. The hematopoietic-specific adaptor protein gads functions in T-cell signaling via interactions with the SLP-76 and LAT adaptors. Curr Biol. 1999;9(2):67-75.

5. Lafont V, Astoul E, Laurence A, Liautard J, Cantrell D. The T cell antigen receptor activates phosphatidylinositol 3-kinase-regulated serine kinases protein kinase B and ribosomal S6 kinase 1. FEBS Lett. 2000;486(1):38-42.

6. Alessi DR, James SR, Downes CP, Holmes AB, Gaffney PR, Reese CB, et al. Characterization of a 3-phosphoinositide-dependent protein kinase which phosphorylates and activates protein kinase Balpha. Curr Biol. 1997;7(4):261-9.

7. Currie RA, Walker KS, Gray A, Deak M, Casamayor A, Downes CP, et al. Role of phosphatidylinositol 3,4,5-trisphosphate in regulating the activity and localization of 3-phosphoinositide-dependent protein kinase-1. Biochem J. 1999;337 ( Pt 3):575-83.

8. Huang Y, Wange RL. T cell receptor signaling: beyond complex complexes. The Journal of biological chemistry. 2004;279(28):28827-30.

9. Thome M, Charton JE, Pelzer C, Hailfinger S. Antigen receptor signaling to NF-kappaB via CARMA1, BCL10, and MALT1. Cold Spring Harb Perspect Biol. 2010;2(9):a003004.

10. Paul S, Schaefer BC. A new look at T cell receptor signaling to nuclear factor-kappaB. Trends Immunol. 2013;34(6):269-81.

11. King CG, Kobayashi T, Cejas PJ, Kim T, Yoon K, Kim GK, et al. TRAF6 is a T cell-intrinsic negative regulator required for the maintenance of immune homeostasis. Nat Med. 2006;12(9):1088-92.

12. Meininger I, Krappmann D. Lymphocyte signaling and activation by the CARMA1-BCL10-MALT1 signalosome. Biol Chem. 2016;397(12):1315-33.

13. Kadamur G, Ross EM. Mammalian phospholipase C. Annu Rev Physiol. 2013;75:127-54.

14. Feske S, Giltnane J, Dolmetsch R, Staudt LM, Rao A. Gene regulation mediated by calcium signals in T lymphocytes. Nat Immunol. 2001;2(4):316-24.

15. Liu JO. Calmodulin-dependent phosphatase, kinases, and transcriptional corepressors involved in T-cell activation. Immunol Rev. 2009;228(1):184-98.

16. Matsuda S, Shibasaki F, Takehana K, Mori H, Nishida E, Koyasu S. Two distinct action mechanisms of immunophilin-ligand complexes for the blockade of T-cell activation. EMBO Rep. 2000;1(5):428-34.

17. Chen ZJ. Ubiquitination in signaling to and activation of IKK. Immunol Rev. 2012;246(1):95-106.

18. Schmidt MH, Dikic I. The Cbl interactome and its functions. Nat Rev Mol Cell Biol. 2005;6(12):907-18.

19. Loeser S, Penninger JM. Regulation of peripheral T cell tolerance by the E3 ubiquitin ligase Cbl-b. Semin Immunol. 2007;19(3):206-14.

20. Thome M. CARMA1, BCL-10 and MALT1 in lymphocyte development and activation. Nat Rev Immunol. 2004;4(5):348-59.

21. Uren AG, O'Rourke K, Aravind LA, Pisabarro MT, Seshagiri S, Koonin EV, et al. Identification of paracaspases and metacaspases: two ancient families of caspase-like proteins, one of which plays a key role in MALT lymphoma. Mol Cell. 2000;6(4):961-7.

22. Lucas PC, Yonezumi M, Inohara N, McAllister-Lucas LM, Abazeed ME, Chen FF, et al. Bcl10 and MALT1, independent targets of chromosomal translocation in malt lymphoma, cooperate in a novel NF-kappa B signaling pathway. J Biol Chem. 2001;276(22):19012-9.

23. Smith-Garvin JE, Koretzky GA, Jordan MS. T Cell Activation. Annual Review of Immunology. 2009;27:591-619.

24. Horejsi V, Zhang W, Schraven B. Transmembrane adaptor proteins: organizers of immunoreceptor signalling. Nat Rev Immunol. 2004;4(8):603-16.

25. Lindquist JA, Simeoni L, Schraven B. Transmembrane adapters: attractants for cytoplasmic effectors. Immunol Rev. 2003;191:165-82.

26. Huang Y, Wange RL. T cell receptor signaling: beyond complex complexes. J Biol Chem. 2004;279(28):28827-30.

27. Joshi RP, Koretzky GA. Diacylglycerol kinases: regulated controllers of T cell activation, function, and development. Int J Mol Sci. 2013;14(4):6649-73.

28. Round JL, Humphries LA, Tomassian T, Mittelstadt P, Zhang M, Miceli MC. Scaffold protein Dlgh1 coordinates alternative p38 kinase activation, directing T cell receptor signals toward NFAT but not NF-kappaB transcription factors. Nat Immunol. 2007;8(2):154-61.

29. Filipp D, Leung BL, Zhang J, Veillette A, Julius M. Enrichment of lck in lipid rafts regulates colocalized fyn activation and the initiation of proximal signals through TCR alpha beta. J Immunol. 2004;172(7):4266-74.

30. Acuto O, Di Bartolo V, Michel F. Tailoring T-cell receptor signals by proximal negative feedback mechanisms. Nat Rev Immunol. 2008;8(9):699-712.

31. Gorska MM, Stafford SJ, Cen O, Sur S, Alam R. Unc119, a novel activator of Lck/Fyn, is essential for T cell activation. J Exp Med. 2004;199(3):369-79.

32. Harada Y, Ohgai D, Watanabe R, Okano K, Koiwai O, Tanabe K, et al. A single amino acid alteration in cytoplasmic domain determines IL-2 promoter activation by ligation of CD28 but not inducible costimulator (ICOS). J Exp Med. 2003;197(2):257-62.

33. Diehn M, Alizadeh AA, Rando OJ, Liu CL, Stankunas K, Botstein D, et al. Genomic expression programs and the integration of the CD28 costimulatory signal in T cell activation. Proc Natl Acad Sci U S A. 2002;99(18):11796-801.

34. Hacker H, Karin M. Regulation and function of IKK and IKK-related kinases. Sci STKE. 2006;2006(357):re13.

35. Lin X, Cunningham ET, Jr., Mu Y, Geleziunas R, Greene WC. The proto-oncogene Cot kinase participates in CD3/CD28 induction of NF-kappaB acting through the NF-kappaB-inducing kinase and IkappaB kinases. Immunity. 1999;10(2):271-80.

36. Hayden MS, Ghosh S. NF-kappaB, the first quarter-century: remarkable progress and outstanding questions. Genes Dev. 2012;26(3):203-34.

37. Wang C, Deng L, Hong M, Akkaraju GR, Inoue J, Chen ZJ. TAK1 is a ubiquitin-dependent kinase of MKK and IKK. Nature. 2001;412(6844):346-51.

38. Togni M, Lindquist J, Gerber A, Kolsch U, Hamm-Baarke A, Kliche S, et al. The role of adaptor proteins in lymphocyte activation. Mol Immunol. 2004;41(6-7):615-30.

39. Czar MJ, Debnath J, Schaeffer EM, Lewis CM, Schwartzberg PL. Biochemical and genetic analyses of the Tec kinases Itk and Rlk/Txk. Biochem Soc Trans. 2001;29(Pt 6):863-7.

40. Raberger J, Boucheron N, Sakaguchi S, Penninger JM, Ellmeier W. Impaired T-cell development in the absence of Vav1 and Itk. Eur J Immunol. 2008;38(12):3530-42.

41. Su B, Jacinto E, Hibi M, Kallunki T, Karin M, Ben-Neriah Y. JNK is involved in signal integration during costimulation of T lymphocytes. Cell. 1994;77(5):727-36.

42. Smith-Garvin JE, Koretzky GA, Jordan MS. T cell activation. Annu Rev Immunol. 2009;27:591-619.

43. Zhang W, Sloan-Lancaster J, Kitchen J, Trible RP, Samelson LE. LAT: the ZAP-70 tyrosine kinase substrate that links T cell receptor to cellular activation. Cell. 1998;92(1):83-92.

44. Werlen G, Hausmann B, Palmer E. A motif in the alphabeta T-cell receptor controls positive selection by modulating ERK activity. Nature. 2000;406(6794):422-6.

45. Palacios EH, Weiss A. Function of the Src-family kinases, Lck and Fyn, in T-cell development and activation. Oncogene. 2004;23(48):7990-8000.

46. Mustelin T, Tasken K. Positive and negative regulation of T-cell activation through kinases and phosphatases. Biochem J. 2003;371(Pt 1):15-27.

47. Bubeck Wardenburg J, Fu C, Jackman JK, Flotow H, Wilkinson SE, Williams DH, et al. Phosphorylation of SLP-76 by the ZAP-70 protein-tyrosine kinase is required for T-cell receptor function. J Biol Chem. 1996;271(33):19641-4.

48. Klein T, Viner RI, Overall CM. Quantitative proteomics and terminomics to elucidate the role of ubiquitination and proteolysis in adaptive immunity. Philos Trans A Math Phys Eng Sci. 2016;374(2079).

49. Satpathy S, Wagner SA, Beli P, Gupta R, Kristiansen TA, Malinova D, et al. Systems-wide analysis of BCR signalosomes and downstream phosphorylation and ubiquitylation. Mol Syst Biol. 2015;11(6):810.

50. Franklin RA, Tordai A, Patel H, Gardner AM, Johnson GL, Gelfand EW. Ligation of the T cell receptor complex results in activation of the Ras/Raf-1/MEK/MAPK cascade in human T lymphocytes. J Clin Invest. 1994;93(5):2134-40.

51. Shambharkar PB, Blonska M, Pappu BP, Li H, You Y, Sakurai H, et al. Phosphorylation and ubiquitination of the IkappaB kinase complex by two distinct signaling pathways. EMBO J. 2007;26(7):1794-805.

52. Chuang HC, Lan JL, Chen DY, Yang CY, Chen YM, Li JP, et al. The kinase GLK controls autoimmunity and NF-kappaB signaling by activating the kinase PKC-theta in T cells. Nat Immunol. 2011;12(11):1113-8.

53. Conner SH, Kular G, Peggie M, Shepherd S, Schuttelkopf AW, Cohen P, et al. TAK1-binding protein 1 is a pseudophosphatase. Biochem J. 2006;399(3):427-34.

54. Kane LP, Mollenauer MN, Xu Z, Turck CW, Weiss A. Akt-dependent phosphorylation specifically regulates Cot induction of NF-kappa B-dependent transcription. Mol Cell Biol. 2002;22(16):5962-74.

55. Hardy K, Chaudhri G. Activation and signal transduction via mitogen-activated protein (MAP) kinases in T lymphocytes. Immunology and cell biology. 1997;75(6):528-45.

56. Cuadrado A, Nebreda AR. Mechanisms and functions of p38 MAPK signalling. Biochem J. 2010;429(3):403-17.

57. Blonska M, Pappu BP, Matsumoto R, Li H, Su B, Wang D, et al. The CARMA1-Bcl10 signaling complex selectively regulates JNK2 kinase in the T cell receptor-signaling pathway. Immunity. 2007;26(1):55-66.

58. Liu Y, Song R, Gao Y, Li Y, Wang S, Liu HY, et al. Protein kinase C-delta negatively regulates T cell receptor-induced NF-kappaB activation by inhibiting the assembly of CARMA1 signalosome. J Biol Chem. 2012;287(24):20081-7.

59. Hayden MS, Ghosh S. Signaling to NF-kappaB. Genes Dev. 2004;18(18):2195-224.

60. Weil R, Israel A. Deciphering the pathway from the TCR to NF-kappaB. Cell Death Differ. 2006;13(5):826-33.

61. Wu CJ, Ashwell JD. NEMO recognition of ubiquitinated Bcl10 is required for T cell receptor-mediated NF-kappaB activation. Proc Natl Acad Sci U S A. 2008;105(8):3023-8.

62. Macian F. NFAT proteins: key regulators of T-cell development and function. Nat Rev Immunol. 2005;5(6):472-84.

63. Gomez del Arco P, Martinez-Martinez S, Maldonado JL, Ortega-Perez I, Redondo JM. A role for the p38 MAP kinase pathway in the nuclear shuttling of NFATp. J Biol Chem. 2000;275(18):13872-8.

64. Yasuda K, Nagafuku M, Shima T, Okada M, Yagi T, Yamada T, et al. Cutting edge: Fyn is essential for tyrosine phosphorylation of Csk-binding protein/phosphoprotein associated with glycolipid-enriched microdomains in lipid rafts in resting T cells. J Immunol. 2002;169(6):2813-7.

65. Park SG, Schulze-Luehrman J, Hayden MS, Hashimoto N, Ogawa W, Kasuga M, et al. The kinase PDK1 integrates T cell antigen receptor and CD28 coreceptor signaling to induce NF-kappaB and activate T cells. Nat Immunol. 2009;10(2):158-66.

66. Porciello N, Tuosto L. CD28 costimulatory signals in T lymphocyte activation: Emerging functions beyond a qualitative and quantitative support to TCR signalling. Cytokine Growth Factor Rev. 2016;28:11-9.

67. Okkenhaug K, Bilancio A, Emery JL, Vanhaesebroeck B. Phosphoinositide 3-kinase in T cell activation and survival. Biochem Soc Trans. 2004;32(Pt 2):332-5.

68. Rameh LE, Cantley LC. The role of phosphoinositide 3-kinase lipid products in cell function. J Biol Chem. 1999;274(13):8347-50.

69. Bonvini E, DeBell KE, Veri MC, Graham L, Stoica B, Laborda J, et al. On the mechanism coupling phospholipase Cgamma1 to the B- and T-cell antigen receptors. Adv Enzyme Regul. 2003;43:245-69.

70. Altman A, Villalba M. Protein kinase C-theta (PKC theta): a key enzyme in T cell life and death. J Biochem. 2002;132(6):841-6.

71. Lee KY, D'Acquisto F, Hayden MS, Shim JH, Ghosh S. PDK1 nucleates T cell receptor-induced signaling complex for NF-kappaB activation. Science. 2005;308(5718):114-8.

72. Villalba M, Bi K, Hu J, Altman Y, Bushway P, Reits E, et al. Translocation of PKC[theta] in T cells is mediated by a nonconventional, PI3-K- and Vav-dependent pathway, but does not absolutely require phospholipase C. J Cell Biol. 2002;157(2):253-63.

73. Avruch J, Khokhlatchev A, Kyriakis JM, Luo Z, Tzivion G, Vavvas D, et al. Ras activation of the Raf kinase: tyrosine kinase recruitment of the MAP kinase cascade. Recent Prog Horm Res. 2001;56:127-55.

74. Di Fiore PP. Signal transduction: life on Mars, cellularly speaking. Nature. 2003;424(6949):624-5.

75. Poltorak M, Meinert I, Stone JC, Schraven B, Simeoni L. Sos1 regulates sustained TCR-mediated Erk activation. Eur J Immunol. 2014;44(5):1535-40.

76. Buday L, Egan SE, Rodriguez Viciana P, Cantrell DA, Downward J. A complex of Grb2 adaptor protein, Sos exchange factor, and a 36-kDa membrane-bound tyrosine phosphoprotein is implicated in ras activation in T cells. J Biol Chem. 1994;269(12):9019-23.

77. Genot E, Cantrell DA. Ras regulation and function in lymphocytes. Curr Opin Immunol. 2000;12(3):289-94.

78. Kortum RL, Rouquette-Jazdanian AK, Samelson LE. Ras and extracellular signal-regulated kinase signaling in thymocytes and T cells. Trends Immunol. 2013;34(6):259-68.

79. Roose JP, Mollenauer M, Gupta VA, Stone J, Weiss A. A diacylglycerol-protein kinase C-RasGRP1 pathway directs Ras activation upon antigen receptor stimulation of T cells. Mol Cell Biol. 2005;25(11):4426-41.

80. Dower NA, Stang SL, Bottorff DA, Ebinu JO, Dickie P, Ostergaard HL, et al. RasGRP is essential for mouse thymocyte differentiation and TCR signaling. Nat Immunol. 2000;1(4):317-21.

81. Salojin KV, Zhang J, Meagher C, Delovitch TL. ZAP-70 is essential for the T cell antigen receptor-induced plasma membrane targeting of SOS and Vav in T cells. J Biol Chem. 2000;275(8):5966-75.

82. Hill CS, Wynne J, Treisman R. The Rho family GTPases RhoA, Rac1, and CDC42Hs regulate transcriptional activation by SRF. Cell. 1995;81(7):1159-70.

83. Li Y, He X, Wang S, Shu HB, Liu Y. USP2a positively regulates TCR-induced NF-kappaB activation by bridging MALT1-TRAF6. Protein Cell. 2013;4(1):62-70.

84. Turner M, Billadeau DD. VAV proteins as signal integrators for multi-subunit immune-recognition receptors. Nat Rev Immunol. 2002;2(7):476-86.

85. Gu JJ, Ryu JR, Pendergast AM. Abl tyrosine kinases in T-cell signaling. Immunol Rev. 2009;228(1):170-83.
